# Domain-general cognitive motivation: evidence from economic decision-making

**DOI:** 10.1186/s41235-021-00272-7

**Published:** 2021-02-04

**Authors:** Jennifer L. Crawford, Sarah A. Eisenstein, Jonathan E. Peelle, Todd S. Braver

**Affiliations:** 1 Department of Psychological and Brain Sciences, Washington University in St. Louis, 1 Brookings Dr, Box 1125, St. Louis, MO 63130 USA; 2 Department of Psychiatry, Washington University in St. Louis, 660 South Euclid Avenue, Box 8225, St. Louis, MO 63110 USA; 3 Department of Radiology, Washington University in St. Louis, 660 South Euclid Avenue, Box 8225, St. Louis, MO 63110 USA; 4 Department of Otolaryngology, Washington University in St. Louis, 660 South Euclid Avenue, Box 8115, St. Louis, MO 63110 USA

**Keywords:** Cognitive motivation, Listening effort, Working memory, Speech comprehension

## Abstract

Stable individual differences in cognitive motivation (i.e., the tendency to engage in and enjoy effortful cognitive activities) have been documented with self-report measures, yet convergent support for a trait-level construct is still lacking. In the present study, we use an innovative decision-making paradigm (COG-ED) to quantify the costs of cognitive effort, a metric of cognitive motivation, across two distinct cognitive domains (working memory and speech comprehension). We hypothesize that cognitive motivation operates similarly within individuals, regardless of domain. Specifically, we test whether individual differences in effort costs are stable across domains, even after controlling for other potential sources of shared individual variation. Conversely, we evaluate whether the costs of cognitive effort across domains may be better explained in terms of other relevant cognitive and personality-related constructs, such as working memory capacity or reward sensitivity.

## Introduction

People frequently make decisions regarding whether to engage in cognitively effortful activities (such as taking on a challenging project at work), or instead, choosing a less effortful alternative (such as mindlessly browsing the internet). Motivation is likely to serve as the key factor impacting the decision to engage in cognitively effortful activities. Indeed, when faced with the choice of whether to engage in a cognitively effortful activity, both the costs (e.g., how taxing is the activity) and benefits (e.g., how much will it improve career prospects) need to be weighed in order to come to a decision. Although cognitive effort-based decision-making likely varies according to the particulars of any given situation, stable individual differences in motivation may also play an important role in the decision-making process. Whereas some individuals might tend to strongly weigh the costs of cognitive effort, choosing to forgo effortful activities more generally, others may welcome the challenges presented to them and engage in a multitude of cognitively demanding tasks in daily life. In other words, people appear to differ in the degree to which they have the motivation to engage with cognitively demanding tasks, or ideas.

Support for this trait-like tendency to engage in cognitively effortful activities has been found in personality psychology research. In particular, the construct of Need For Cognition, assessed via self-report questionnaire (Need for Cognition Scale; NCS), is conceptualized as a stable individual difference in the tendency to engage in, and enjoy, effortful cognitive activities (Cacioppo et al. 1996; Cacioppo and Petty 1982), and is often referred to with the short-hand terminology of “cognitive motivation”. Likewise, individual differences in cognitive motivation, assessed via the NCS, have been found to relate to important life outcomes, such as academic achievement and the ability to seek out and scrutinize information pertinent to daily decision-making (Cacioppo et al. 1996).

Taking a closer look at this definition, it is important to note the critical distinction between an individual’s need for cognition (i.e., cognitive motivation) and their cognitive abilities. Cognitive ability is well-established as a major dimension of individual variation, and is assessed both through general intelligence tests (e.g., standard IQ measures), as well as more specialized dimensions, such as fluid intelligence (which indexes novel problem-solving and reasoning ability) and working memory capacity (which indexes the degree to which information can be actively maintained in short-term storage and used towards on-going cognitive computation). Nevertheless, cognitive motivation has been conceptualized as a trait that operates distinctly from cognitive ability (Cacioppo et al. 1996), suggesting that it is a meaningful and unique construct in the study of individual differences. Indeed, empirical work supports this claim, demonstrating that an individual’s cognitive motivation is related to, but distinct from, their fluid intelligence (Fleischhauer et al. 2010; Hill et al. 2013) and working memory capacity (Hill et al. 2016; Therriault et al. 2015). Taken together, these findings provide support for the claim that cognitive motivation is a domain-general construct that indexes the propensity of an individual to engage in cognitively effortful activities independent of their cognitive and intellectual abilities.

Nevertheless, our current understanding of individual differences in cognitive motivation is constrained by limitations in the way that this construct has typically been assessed. Specifically, individual differences in cognitive motivation are almost exclusively reported using self-report measures, like the NCS (Cacioppo and Petty 1982). Self-report measures have a number of well-recognized limitations, such as memory-related biases in retrospective reporting, susceptibility to demand characteristics, and social desirability concerns (Barrett et al. 1998). As a consequence of these well-recognized limitations, recent work has shifted the focus of investigation from self-report measures to sensitive behavioral indices of cognitive motivation using methods from the field of behavioral economics. More specifically, these new developments place cognitive motivation within a decision-making framework in which cognitive motivation is measured using revealed preferences, reflecting the trade-off between the expected benefits and costs associated with engaging in cognitively effortful activities (Botvinick and Braver 2015; Shenhav et al. 2013; Westbrook and Braver 2015). For example, decision-making paradigms, such as the Demand Selection Task (DST; Kool et al. 2010), have enabled the precise quantification of cognitive motivation using revealed preferences between performing tasks with more, or less, frequent task-switching, rather than using explicitly stated preferences; this work has demonstrated that individuals tend to avoid engaging in cognitive effort (Kool and Botvinick 2014; Kool et al. 2010).

Similar considerations have prompted the development, within our own group, of a novel decision-making paradigm known as the COG-ED (for Cognitive Effort Discounting task) (Westbrook et al. 2013). The COG-ED derives from other well-known discounting paradigms used in behavioral and neuroeconomics that have been used to examine how other cost factors such as delay, risk or physical effort impact decision-making regarding reward outcomes (Green and Myerson 2013). For example, the EEfRT (Effort Expenditure for Rewards Task; Treadway et al. 2009) is a widely used physical effort-based decision-making task that has been shown to be sensitive to individual differences (Treadway et al. 2012a, b) and clinical impairments, such as schizophrenia and depression (Barch et al. 2014; Treadway et al. 2012a, b). Like the COG-ED, these tasks use decision-making trials to estimate the “point of subjective indifference” (or equivalence), in which two options are equally preferred, which can be used to determine how much a particular cost factor “discounts” the value of a given outcome. For example, in delay discounting paradigms, if an individual is found to equally prefer receiving $10 now to $25 in a month, then the 1-month delay is estimated to discount the reward value by $15.

In the COG-ED, the focus is on cognitive effort discounting, as participants make a series of decisions between low-effort, low-reward and high-effort, high-reward options to identify their point of subjective indifference (Westbrook et al. 2013). The COG-ED has shown to be sensitive to individual differences in cognitive motivation: individuals higher in cognitive motivation, as indexed by the NCS, tend to choose performing cognitively effortful tasks more often than those with lower levels of cognitive motivation (Westbrook et al. 2013). Most recently, the COG-ED has also been examined in the domain of speech comprehension, to test the degree to which subjective effort is increased when trying to understand speech in the midst of background noise (McLaughlin et al. 2020). Both young and older adults discounted effortful listening, and in older, but not young adults, this was tied to both hearing ability and working memory capacity. Moreover, age differences in effortful listening still remained even when accounting for these ability factors, consistent with a role for cognitive motivation. Thus, the COG-ED offers a promising tool to test whether cognitive motivation operates as a trait-like construct across task domains and individuals.

Importantly, it has still not been rigorously tested whether the extant findings from the COG-ED and related behavioral economic paradigms reflect stable individual differences in the specific construct of cognitive motivation, rather than individual differences in other constructs, such as cognitive ability or other personality-related motivational indices (e.g., reward sensitivity). Furthermore, to date our understanding of individual differences in cognitive motivation has been limited due to testing this construct in just one task domain at a time. Thus, in order to more carefully test whether cognitive motivation indeed operates at a domain-general level, individual preferences need to be tested across multiple domains in order to de-confound them from the processes that underlie the cognitive tasks themselves, such as working memory capacity. Indeed, recent work has attempted to remedy these gaps in our understanding by assessing cognitive motivation across two different versions of the DST, in addition to collecting individual difference measures of cognitive motivation (e.g., NCS) and ability (e.g., Trail Making Test; Strobel et al. 2020). Interestingly, this study found that both the behavioral and self-reported measures of cognitive motivation showed evidence of trait variance when controlling for cognitive ability; however, the two measures did not correlate with each other (Strobel et al. 2020). On the surface, these results seem to suggest that the behavioral paradigms aimed at assessing cognitive motivation do not map onto measures indexing the same construct via self-report. However, since this experiment only tested one type of economic decision-making paradigm (DST), the results leave open the possibility that the null findings reflected the particular paradigm used, and that other decision-making paradigms, such as the COG-ED, may provide more robust indices of the latent cognitive motivational construct.

Following up from this recent work, in the current study we aim to test whether individual differences in participants’ cognitive motivation show strong relationships across distinct cognitive domains. More specifically, by using the COG-ED to quantify cognitive effort costs (in addition to assessment with the more traditional NCS), we will examine whether individuals who exhibit high cognitive motivation, within the domain of working memory, also exhibit high cognitive motivation in the domain of speech comprehension. Thus, we will assess cognitive motivation in two distinct domains, both of which rely on some of the same cognitive processes (Peelle 2018), to test whether cognitive motivation is a stable, domain-general trait that can be observed across multiple cognitive contexts, using a sensitive behavioral paradigm. Indeed, we predict that we will observe a strong association between the costs of cognitive effort (i.e., cognitive motivation) in working memory and speech comprehension domains, suggesting that there is a stable, trait-like, cognitive motivational construct that contributes to an individual’s cognitive effort costs (hypothesis 1). Moreover, even when controlling for other relevant processes (e.g., working memory capacity, personality traits indexing reward motivation), we predict that there will still be an association between cognitive effort costs across working memory and speech comprehension domains, providing stronger evidence for a domain-general cognitive motivational construct (hypothesis 2).

## Methods

### Ethics information

All experimental procedures will be approved by the Washington University Human Research Protections Office prior to data collection. Participants will provide informed consent and will be compensated $10/h for all study procedures, with the opportunity to gain up to an additional $8 bonus, based on the experimental tasks.

### Pilot data

A sample of healthy adults (*N* = 31, 18–23 years old) completed a pilot study to assess the feasibility of completing cognitive effort discounting procedures across both working memory and speech comprehension domains (see Additional file 1 for further details). As a brief overview, participants completed a task familiarization phase in which they performed either a N-back task, with working memory load varied across blocks (i.e., how many previous items need to be stored in working memory; *N* = 1–4, with higher *N* indicating increased cognitive demands), or a speech-in-noise task, with effortful speech comprehension varied across blocks (i.e., listening to spoken sentences presented with different levels of background noise; signal-to-noise ratios [SNRs] ranging from − 12 to 0 dB, with lower numbers corresponding to greater cognitive demands). Following the familiarization phase, participants completed a decision-making phase, by performing the COG-ED in each of the two domains (i.e., N-Back, speech-in-noise). In the COG-ED, with conditions adapted from prior work (Westbrook et al. 2013), participants were required to make a series of decisions between performing high-effort task levels (e.g., 2–4 back; -4,-8, -12 SNR) for high monetary reward or low-effort task levels (e.g., 1-back; 0 SNR) for a lower monetary reward value. Critically, a within-subject design was employed, with each participant completing both the familiarization and discounting phases in both working memory and speech comprehension domains (counterbalanced across participants). This design enabled us to quantify the subjective costs of cognitive effort for each participant in each domain, and to look at relationships between them.

**Fig. 1:**
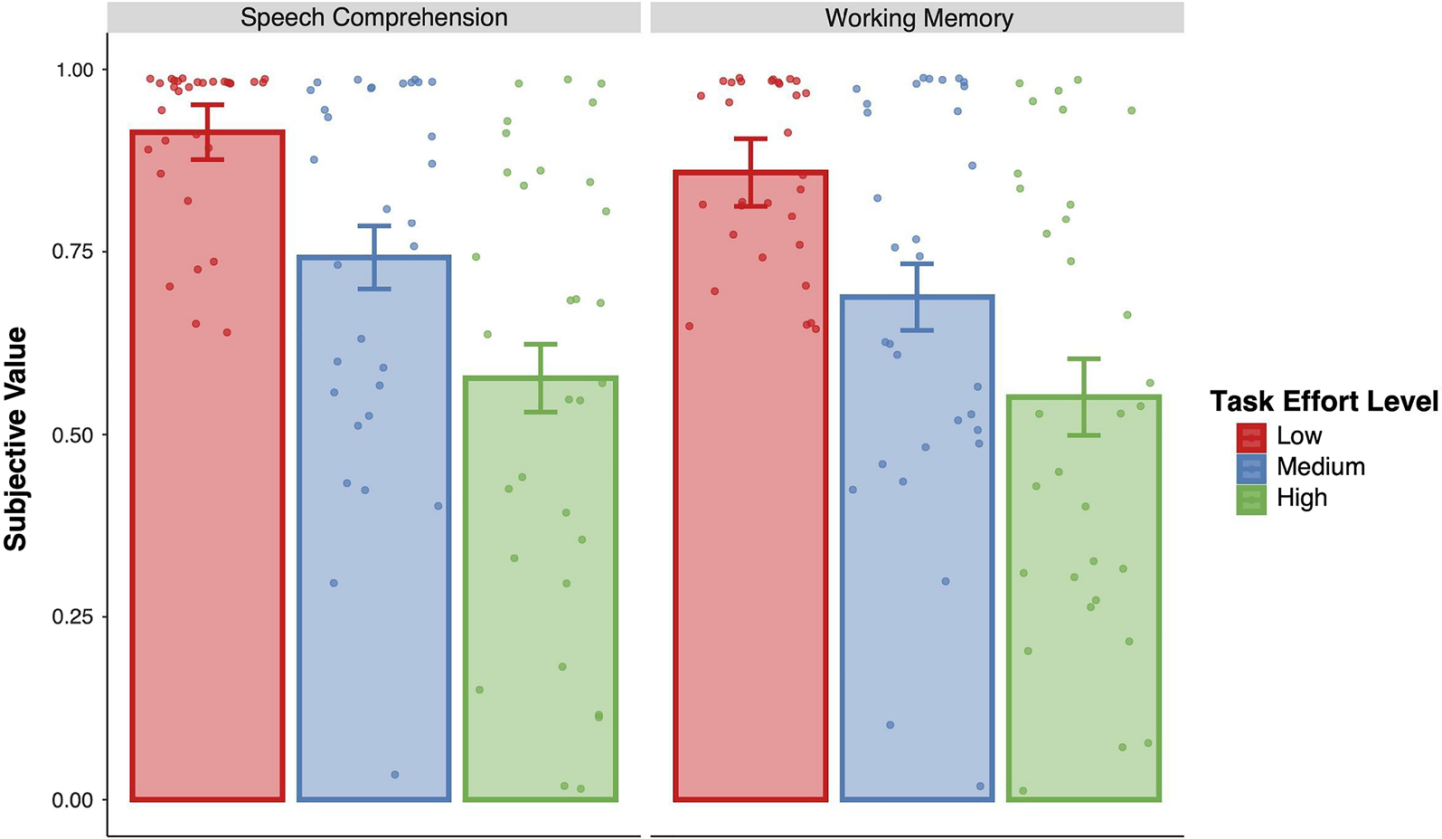
Effects of task load (low effort: 2-back, -4 SNR; medium effort: 3-back, -8S NR; high effort: 4-back, -12 SNR) on subjective value estimates in working memory and speech comprehension domains. Error bars represent 95% CIs

**Fig. 2:**
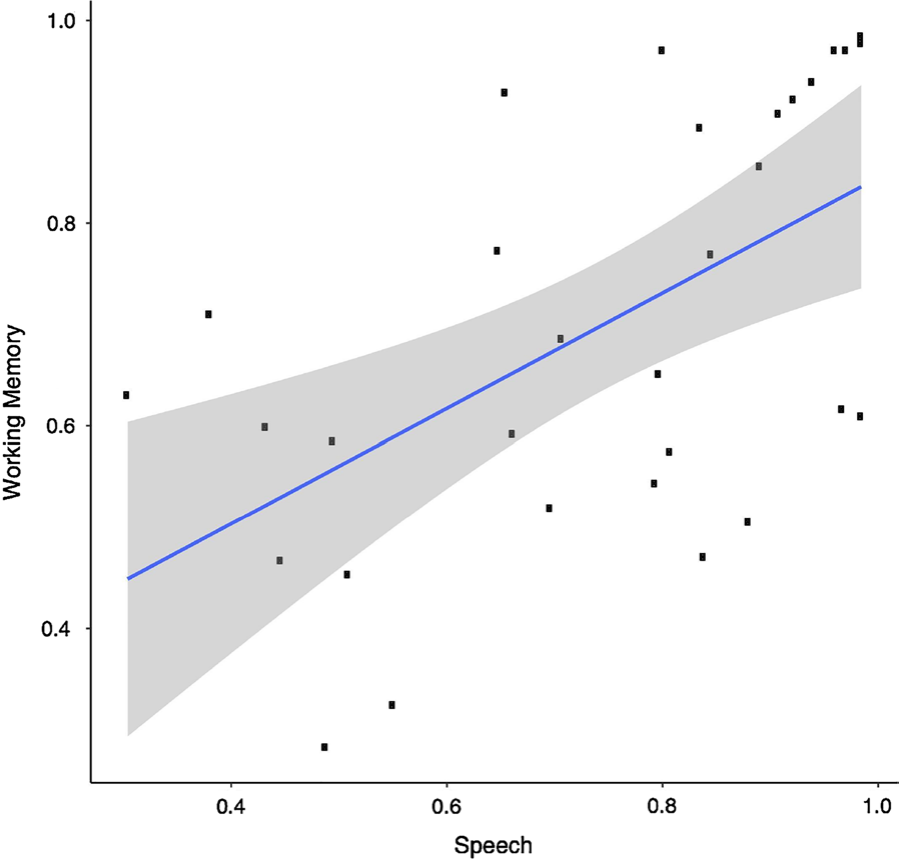
Correlation of average subjective value estimates across working memory and speech domains

We found that across both domains, participants discount task load (i.e., cognitive effort) similarly, whereby more difficult levels of the task (i.e., purple; 4-Back, -12 SNR) are discounted more, or have a lower subjective value, relative to easier task levels (i.e., red; 2-Back, -4 SNR), *β* = -0.15 [− 0.12, − 0.18], SD = 0.02, with no differences observed across domains, *β* = 0.08 [− 0.02, 0.17], SD = 0.05 (Fig. 1). Furthermore, examining the average subjective value of cognitive effort across working memory and speech domains reveals a strong association of the costs of cognitive effort within-subject, *r* = 0.521 [0.234, 0.744], BF_10_ = 39.21. In other words, participants who exhibited a low subjective value of cognitive effort (i.e., find engaging in cognitive effort to be costlier) in the working memory domain, also tend to have a low subjective value of cognitive effort in the speech comprehension domain (Fig. 2). The relationship between the costs of cognitive effort in working memory and speech comprehension domains remained, even after controlling for individual differences related to task difficulty and performance in each respective domain (working memory: d-prime, mean RT; speech comprehension: intelligibility), *r* = 0.3997 [0.213, 0.558], BF_10_ = 34.1.

Self-reported ratings of mental demand, effort, and frustration provide further support of the costs of cognitive effort in each domain. There was a main effect of task load across ratings of mental demand *β* = 13.95 [11.09, 16.73], SD = 1.43, effort *β* = 11.81 [9.09, 14.49], SD = 1.38, and frustration *β* = 8.49 [5.68, 11.31], SD = 1.43. This suggests that as task load level increased, subjective ratings of mental demand, effort, and frustration increased. However, in contrast to the behavioral findings, there was also a main effect of domain for self-reported ratings of effort *β* = − 15.81 [− 23.23, − 8.23], SD = 3.79, and mental demand *β* = − 10.37 [− 17.50, − 3.19], SD = 3.65, which demonstrates that participants rated the speech-in-noise task to be less mentally demanding and effortful overall, relative to the working memory task. Frustration ratings did not differ across task domain, *β* = − 0.39 [− 7.82, 7.00], SD = 3.82.

Furthermore, we did not find conclusive evidence for a relationship between self-reported (e.g. NCS) and behavioral measures of cognitive motivation (e.g., cognitive effort discounting) in the pilot sample. Correlations between NCS and the working memory COG-ED (*r* = 0.115 [− 0.218, 0.0.451], BF_10_ = 0.33), speech comprehension COG-ED (*r* = 0.174 [− 0.145, 0.493], BF_10_ = 0.45), and the composite COG-ED score (*r* = 0.158 [− 0.197, 0.471], BF_10_ = 0.42) were anecdotal. It is important to note that in the pilot data, other potential covariates, such as working memory capacity or personality traits, were not assessed.

### Design

To examine the relationship between lab-based measures of cognitive effort, we will use the COG-ED (Westbrook et al. 2013) to estimate the subjective value (i.e., cost) of cognitive effort across two domains (i.e., working memory, speech comprehension) and test for associations between the subjective value of cognitive effort, within-subject. Moreover, we will obtain individual difference measures of the component processes that are most likely to contribute to the computation of the cognitive effort costs (i.e., working memory capacity, reward sensitivity) in order to control for their influence when assessing the strength of the association of cognitive effort discounting across working memory and speech comprehension domains.

Orthogonal to our main hypotheses of interest, we will also collect a self-reported measure of cognitive motivation (NCS), in order to test for the strength of the association between self-reported and behavioral indices of cognitive motivation. Although this doesn’t fall within the primary scope of this experiment, collecting these data will provide an important baseline of research needed to rigorously explore the relationships between self-reported and behavioral measures of cognitive motivation in future work.

The experiment will take place via on-line testing, across two separate sessions, scheduled on different days. In the first experimental session, participants will be assessed on a range of individual difference measures, that index working memory capacity (Listening-span; L-span; Cai et al. 2015; Operation-Span; O-Span; Symmetry-Span; Sym-Span; Unsworth et al. 2005). In addition we will collect self-report measures of reward motivation: Behavioral Inhibition and Behavioral Activation Scales (BIS/BAS; Carver and White 1994), Generalized Reward and Punishment Expectancy Scale (GRAPES; Ball and Zuckerman 1990), and Sensitivity to Punishment and Sensitivity to Reward Questionnaire (SPSRQ; Torrubia et al. 2001). Self-reported cognitive motivation (NCS; Cacioppo and Petty 1982) will also be collected for use with exploratory analyses. All tasks and questionnaires during this session will be administered in the same order across participants.

In the second experimental session, participants will complete the familiarization and decision-making phases of the COG-ED within each cognitive domain. During the familiarization phase, participants will first experience variously demanding levels of either a working memory or speech-in-noise task; task order will be fixed across participants. During the working memory task (i.e., N-Back), a sequence of letters is presented one at a time in the center of a computer screen. The task requires that participants indicate when the current stimulus (i.e., letter) matches the letter from *N* steps earlier in the sequence (target) or when the stimulus differs from the letter presented *N* steps earlier (non-target). Prior work has shown that as the level of N increases, the task becomes progressively more difficult and effortful (Ewing and Fairclough 2010). Participants will complete one 64-trial run (16 targets; 48 non-targets) of each level of the task (*N* = 1–4) in ascending order of difficulty. Each level of the task is assigned a color (i.e., 2-Back = “red”) to avoid anchoring effects (i.e., cognitive biases that could cause subjects to base judgments off of an initial (or baseline) level of difficulty; Ariely et al. 2006). Thus, participants will learn to associate each task level with its assigned color before beginning the discounting procedure. This discounting procedure has been successfully used across multiple participant populations, showing robust effects (Culbreth et al. 2019; Westbrook et al. 2013).

During the speech-in-noise task, adapted from McLaughlin et al. (2020), participants will be presented with sentences with varying levels of noise. Prior to starting the experiment, participants will be encouraged to locate to a quiet space and use headphones for the task, if possible. The signal-to-noise ratio (SNR) will be adjusted to manipulate task difficulty; negative SNR values indicate that the signal is presented at a lower level than the noise. Sentences will be presented at various levels of noise (i.e., 0 dB SNR, − 12 dB SNR), and participants will be instructed to type the sentence they heard back into a text box on each trial. Participants are instructed to guess if they were unsure of any words in a sentence. Each task level consists of 16 self-paced trials wherein participants will hear a sentence, type it back into a text box, and then use the spacebar to begin the next trial. Like the working memory task, participants will complete task blocks in order of difficulty, from easiest (i.e., 0 SNR) to hardest (i.e., − 12 SNR), with the same color mappings for task difficulty used in the working memory task. Both familiarization blocks (working memory, speech comprehension) are roughly equated in total duration.

Following each run of the familiarization task (e.g., completing the 1-Back or 0 SNR task), participants will complete self-reported ratings of the mental demand, physical demand, temporal demand, effort, frustration, and performance from the preceding task block using the NASA Task Load Index (Hart 2006). Participants will provide their responses using a visual analog scale ranging from 0 (very low) to 100 (very high). These ratings will serve as a manipulation check to ensure that participants find the tasks to be effortful and mentally demanding across each load level.

After the familiarization phase, in which each load level is experienced and practiced, the critical decision-making phase of the COG-ED occurs. In this phase, participants make repeated choices about whether to repeat performance of a higher load-level of the task (e.g. 4-back, -12 SNR) or instead perform the easiest load level (1-back, 0 SNR). The first trial of each higher- and low-effort pairing will present participants with equal reward amounts (either $2, $3, or $4) for completing the chosen task (e.g., $2 for 1-back vs. $2 for 2-back).The offer for the chosen task is then stepwise titrated until participants are indifferent between the two offers (i.e., they would choose either offer equally). For example, if a participant chose the $2 for 1-Back, over $2 for the 2-Back, then the next calibration trial would present the participant with the offer of performing the 1-Back for $1 (i.e., half of the amount of the previous offer) or performing the 2-Back for $2 (i.e., fixed offer amount). On the other hand, if the participant instead chose to perform the 2-Back for $2 on the first trial (relative to $2 for the 1-Back) then the offer amount for the higher effort option would be stepwise titrated until the indifference point is reached. The point of subjective indifference is critical because it quantifies how much more subjectively costly the unchosen task level is relative to the chosen task. As a result, these indifference points estimate the “cost” of cognitive effort. In other words, the indifference point is the amount of money an individual is willing to forgo to avoid performing the unchosen task.

Participants will complete a total of 45 decision trials in each domain (3 task levels × 3 reward levels × 5 calibration trials) after they complete the corresponding familiarization phase. Critically, participants will be informed that one of their choices will be used to determine task-based compensation and that they will be asked to repeat the task they chose, for the amount of money offered (i.e., $2 for the “red” task). Task-based compensation is not based on performance from the familiarization phase, but rather, participants will be told that in order to successfully earn the money for repeating the chosen task, they need to maintain their effort from the familiarization block when repeating the task block.

In addition, after completing all task blocks in each domain, participants will be asked to complete a post-task questionnaire to assess how much their choices during the discounting phase were based on the difficulty, effort, or monetary reward associated with the task. In addition, after completing the speech comprehension phase, participants will be asked what device was used to complete the task (e.g., speakers, headphones). A complete description of all self-report questionnaires is provided in the Additional file 1. Data collection and analysis will not be performed blind to the conditions of the experiments.

### Sampling Plan

Participants will be healthy adults, ages 18–40, recruited through the online research platform Prolific (www.prolific.co) (Palan and Schitter 2018). Participants will be excluded if they are not native English speakers, have current or previous history of neurological trauma, seizures, hearing difficulty, or mental illness, report current use of psychotropic medications, or report not using headphones during the speech comprehension task. We will strive to use all available data in the subsequent analyses. However, if an individual appears to present with behavioral patterns that suggest non-compliance with the task instructions (e.g., always choosing the high-effort option), we will perform supplemental data analysis both with and without the excluded participant(s) and report both sets of values. Furthermore, if technical difficulties arise during data collection that prevent either of the cognitive effort discounting procedures from being recorded, or if a participant withdraws from the study prematurely, the data from that participant will not be used in subsequent analyses.

### Power analysis

We used Bayes Factor Design Analysis (BFDA) to determine the sample size for this experiment. Adopting a sequential design with maximal N using BFDA will help to ensure that we are collecting sufficient evidence while maintaining efficiency in our design (Schönbrodt and Wagenmakers 2018; Schönbrodt et al. 2017). As an overview, in sequential designs, sampling is continued until the desired level of the strength of evidence is reached (i.e., Bayes factor; BF_10_), which in this case is 10 times in favor of the experimental hypothesis over the null hypothesis, or vice versa. To strike a balance between the feasibility and interpretability of the results, we will stop all data collection after the maximal N for this study (*N* = 300) has been collected, if the Bayes Factor threshold has not already been reached. To aid in the calculation of the approximate sample size, we used the BFDA app (http://shinyapps.org/apps/BFDA/; Stefan et al. 2019), which runs 10,000 Monte Carlo simulations based on the pre-specified prior distribution and effect size estimates provided by the user. For this experiment, we opted to follow the approach of a safeguard power analysis (Perugini et al. 2014), choosing a smaller effect size (*r* = 0.3) than what was previously observed in our pilot study (*r* ~ 0.5 or *r* ~ 0.4 after controlling for task performance) in order to avoid underestimating the sample size. Furthermore, we decided to use an uninformed prior, a central Cauchy distribution with a scaling parameter of *r* = √2/2, as is default in the BayesFactor (Morey and Rouder 2015) package in R, taking a more conservative approach to power analysis.

Results from the simulations suggest that the median sample size needed to obtain a Bayes factor ≥ 10 given the parameters specified above is *N* = 112, and, conversely, finding evidence in support for the null hypothesis, BF_10_ ≤ 0.1, would require a median sample size of *N* = 140 (results summarized in Additional file 1^1^. Thus, we plan to sample, at minimum, 100 participants; after reaching this sample size, we will test for sufficient evidence every ten participants thereafter, until the Bayes factor threshold (i.e., BF_10_ ≥ 10 or BF_10_ ≤ 0.1) is reached or until we have collected data from 300 participants, the maximal *N*.

### Analysis plan

The main variable of interest is the subjective value (i.e., cost) of cognitive effort. The subjective value is calculated using each participant’s responses during the discounting procedure; as an overview, participants make repeated choices between high- and low-effort tasks, each at equal offer amounts at fixed values ($2, $3, $4), and the monetary values of the chosen option (either high- or low-effort task) are then step-wise titrated using each participant’s prior responses. The value of the titrated reward at the end of the task, provides the indifference point (i.e., the value at which the participant is equally likely to choose either the low- or high-effort option). For task choices following trials in which participants initially chose the low-effort option (e.g., discounting high-effort option), each indifference point is divided by the corresponding monetary value of the high-effort option either $2, $3, or $4, to summarize the subjective value of engaging in cognitive effort, a positive value ranging from 0 to 1. If participants initially choose the high-effort option when presented with equal monetary rewards for performing the high- or low-effort task (e.g., discounting low-effort option), we will subtract the indifference point from the fixed monetary reward amount and divide by the value of the fixed monetary reward. We will transform all subjective value estimates in which participants initially chose the high-effort option by adding 1 to the estimate, such that the subjective value estimate will range from 0 to 2; values > 1 indicate preferences for higher effort tasks, whereas values < 1 indicate preference for the easy task.

In the first stage of analysis, we will determine the zero-order correlation between cognitive effort discounting, estimated separately from the working memory and speech comprehension domains. For this analysis, we will first calculate the average subjective value across all task levels for each participant in each domain, then using the BayesFactor (version 0.9.12-4.2; Morey and Rouder 2015) package in R, we will correlate those two subjective value estimates with each other. For this analysis, we will use an uninformed prior, Cauchy distribution (*µ* = 0, *r* = √2/2) and report the correlation value as the median of the posterior distribution, in addition to the 95% credible intervals. Further, we will report the Bayes factor, which contrasts the strength of the experimental model (i.e., correlation between effort costs across domains), relative to the null hypothesis, (i.e., no correlation between effort costs across domains). This analysis will serve to replicate the initial finding in our pilot sample, which showed a strong association between the subjective value of cognitive effort across working memory and speech comprehension domains.

In the second stage of analysis, we will first statistically control for task difficulty and performance in each respective domain prior to computing the correlation between cognitive effort discounting in working memory and speech comprehension domains. To accomplish this, we will enter task-level and relevant task performance variables (N-Back: d-prime, meant RT; Speech: intelligibility) as covariates in a model predicting subjective value in each domain separately; the residuals from each model will be correlated with each other using the same uninformed prior distribution as detailed above in order to quantify the strength of the relationship between effort discounting across domains. This analysis will help to ensure that we are accounting for task-specific variables, such as performance, that could influence the subjective value of cognitive effort across domains.

To extend the results of our pilot study, we will then perform a third stage of analysis that aims to additionally control for the influences of trait-level individual differences in working memory capacity and reward sensitivity when examining the association between the subjective value of cognitive effort across working memory and speech comprehension domains. For working memory capacity, we will create a composite score, for which we will sum the z-scores from the total score from each working memory measure (L-span, O-Span, Sym-Span). Reward sensitivity will be calculated by summing the z-scores obtained in each reward sensitivity measure (BAS total score, GRAPES reward expectancy score, and the SPSRQ reward sensitivity score). These two composite variables (working memory capacity, reward sensitivity) will then serve as covariates in a partial correlation analysis that uses the cognitive effort discounting residual scores estimated for the second stage of analysis. We will use the same uninformed prior distribution as detailed above, to measure the strength of the relationship between the subjective value (i.e., costs) of cognitive effort between working memory and speech comprehension domains, when controlling for the two individual difference measures.

This third-stage of analysis will be critical in determining whether there is a domain-general motivational construct that reflects the costs of cognitive effort, controlling for other relevant processes; if this relationship exists, it would suggest that cognitive motivation can be indexed as a trait-like measure, such that measuring the subjective value of cognitive effort in one domain (i.e., working memory), would predict that an individual exhibits similar behavior in other cognitive domains. In contrast, if the first hypothesis (a correlation between indifference points across the two effort discounting tasks) is confirmed, but the second hypothesis (a persistent correlation with added covariates) is disconfirmed, we will conclude that cognitive motivation is domain-specific. In other words, it is an individual’s working memory capacity and/or, reward sensitivity that accounts for the relationship between the costs of cognitive effort across multiple cognitive (working memory, speech) domains. If the results from the third stage of analysis are inconclusive, we will state that we cannot draw further conclusions regarding whether trait-level individual differences, such as working memory capacity and reward sensitivity, can account for the relationship between the subjective cost of cognitive effort across domains.

## Supplementary Information

**Additional file 1**. Supplemental Information.

## Data Availability

All relevant experimental scripts, data, code, and analyses are located in an online repository on the Open Science Framework: https://osf.io/9t6q7/?view_only=cc143ba2834c4aef8f034ba046b01098.
